# The Impact of Therapeutic Ultrasound on Bone Radio Density Following Orthodontic Treatment with Clear Aligners: A Preliminary Study

**DOI:** 10.2174/0115734056371884250324151755

**Published:** 2025-04-17

**Authors:** Mohsen Gholizadeh, Hollis Lai, Lindsey Westover, Tarek El-Bialy

**Affiliations:** 1 Faculty of Medicine and Dentistry, Mike Petryk School of Dentistry, University of Alberta, Edmonton, Alberta, Canada; 2 Department of Mechanical Engineering,University of Alberta, Edmonton, Canada; 3 Department of Biomedical Engineering, University of Alberta, Edmonton, Canada; 4 Division of Orthodontics, University of Manitoba, Canada

**Keywords:** Low-Intensity Pulsed Ultrasound (LIPUS), Clear Aligners, Bone Radio Density, Hounsfield Units, CBCT, Orthodontic outcomes

## Abstract

**Objective:**

This study evaluated the impact of Low-Intensity Pulsed Ultrasound (LIPUS) on bone radio density in patients undergoing orthodontic treatment with clear aligners, aiming to enhance bone remodeling and improve treatment stability.

**Methods:**

This retrospective study included 68 participants divided into two groups: 34 treated with LIPUS and 34 in a control group. Bone radio density was measured using Hounsfield units from CBCT scans before and after treatment. Statistical analyses included Mann-Whitney U tests and paired *t*-tests.

**Results:**

The average age was 29.85 ± 14.85 years in the control group and 36.29 ± 12.78 years in the LIPUS group. Bone radio density in the upper arch of the LIPUS group significantly increased from 444.6 HU to 751.3 HU (p < 0.001), while the control group showed a slight decrease in the upper arch (657.4 HU to 650.5 HU, p = 0.86). In the lower arch, a similar trend was observed in the LIPUS group, with an increase from 767.7 HU to 823.4 HU (p = 0.17), though not statistically significant. There were no significant differences in post-treatment ABO DI scores between groups, suggesting equivalent effectiveness in achieving orthodontic outcomes.

**Conclusion:**

LIPUS with clear aligners seems promising in enhancing bone radio density, indicating an improved bone remodeling effect. This highlights LIPUS's potential as a beneficial adjunct in orthodontic treatments.

## 1 INTRODUCTION

Creating aligners on setup casts for orthodontic tooth movement was first introduced in 1945 [1]. This innovation was primarily motivated by the growing demand for invisible braces and aesthetic considerations, especially among adult patients. By the late 1990s, two thermoplastic aligner systems were introduced, supporting a broad spectrum of tooth position adjustments [2]. The first system used setups that included tooth displacements with three aligners required for each setup step. The second system introduced fewer setup steps using more rigid aligners [3]. Implementations of stereolithographic models and digital setups eliminated the need for more than one initial impression. These methods gained popularity, particularly among adult patients, spurred by vigorous marketing efforts by the manufacturers [3]. Despite these advancements, significant discrepancies persist between predicted treatment outcomes and actual results, often necessitating multiple refinements and adjustments [4]. This has raised concerns about the efficiency of clear aligners [5].

Low-intensity pulsed ultrasound (LIPUS) technology has emerged as a potential solution to enhance the efficacy of orthodontic treatment with clear aligners by accelerating tooth movement with fewer refinements [6]. LIPUS has been employed in the medical field for over six decades, with applications ranging from sports medicine and physiotherapy to healing fractured non-union bones [7]. LIPUS generates acoustic pressure waves that traverse living tissues, resulting in micromechanical strains and triggering a cascade of molecular events [8]. Studies have shown the effect of LIPUS on reducing orthodontically induced tooth root resorption (OITRR), accelerating orthodontic tooth movement, and shortening orthodontic treatment duration by modulating the expression of key molecules like collagen type 1 (Col1), alkaline phosphatase (ALP), osteoprotegerin (OPG), and receptor activator of nuclear factor-kappa β-ligand (RANK-L) [9-14].

Cone beam computed tomography (CBCT) is pivotal in orthodontics for its detailed three-dimensional imaging capabilities. It is crucial for evaluating dentition and identifying anomalous structures often missed by conventional two-dimensional radiography [15, 16]. CBCT also plays a critical role in measuring bone radio density, which is essential for planning orthodontic treatments and dental implants. Advances in CBCT technology allow for detailed and quantitative bone radio density measurements, providing a reliable method for assessing bone health in orthodontic patients [15, 17-21].

The stability of the final position after orthodontic treatment is a challenge after orthodontic treatments [22]. In the modern era of accelerated orthodontic treatments, concerns persist about the effects of rapid tooth movement on bone density during the retention phase. Research on this topic has shown mixed findings, with some studies indicating a decrease, some an increase, and others no change in bone density following orthodontic treatment [17, 23-25]. This variability may be due to different bone remodeling responses to the type and extent of movement.

LIPUS has shown promise in enhancing bone density by accelerating bone remodeling and stimulating osteoblastic activity, potentially reducing relapse and enhancing the stability of teeth during the retention phase [26]. However, more comprehensive and controlled studies are needed to validate these findings. This study fills a gap in the existing literature by analyzing bone radio density changes with LIPUS, a subject that has not been extensively explored, particularly in orthodontics.

Yu *et al*. (2016) found that orthodontic treatment, particularly with clear aligners, can lead to changes in alveolar bone density, which can affect treatment outcomes and lead to relapse if not properly managed [23]. Similarly, Zhuang *et al*. (2011) reported that bone density may decrease after orthodontic tooth movement, highlighting the potential risk of post-treatment instability [25]. Rinchuse *et al*. (2007) emphasized that the stability of treatment results, particularly during the retention phase, is critical for long-term success [22]. Zheng *et al*. (2024) demonstrated that LIPUS could enhance osteogenesis in periodontal ligament cells under mechanical stress, potentially accelerating the orthodontic treatment and improving bone quality [27]. Moreover, studies by Pascoal *et al*. (2024) have indicated that LIPUS stimulation boosts the viability and proliferation of osteoblasts and periodontal ligament fibroblasts, which are essential for bone repair and remodeling during orthodontic treatment [28].

In orthodontics, a critical challenge is maintaining stable tooth positioning after treatment, as bone remodeling plays a significant role in treatment predictability and long-term results. This study attempts to address this issue by exploring the impact of LIPUS on bone radio density during clear aligner treatment. By providing evidence of LIPUS's effects on bone density, this study offers a potential solution to the longstanding issue of bone resorption and relapse risk post-treatment.

Objectives: This study investigated the effect of LIPUS on bone radio density in patients who underwent orthodontic treatment with clear aligners, focusing on the potential benefits of enhancing bone remodeling and stability. The novelty of this research lies in its examination of how LIPUS may provide a solution to the common issue of bone density changes after orthodontic treatments, which has been overlooked in previous studies. This investigation will contribute to a better understanding of LIPUS's role in improving the predictability and stability of orthodontic outcomes, offering potential clinical benefits for patients.

## 2 MATERIALS AND METHODS

This retrospective analysis focused on the patient records (from 2019 to 2024) from a private orthodontic clinic in Edmonton, Alberta. The patients underwent orthodontic treatment with Invisalign™ clear aligners, with or without LIPUS. The Aevo System (model A3) was the device that applied LIPUS. The study sought to identify if there was any difference between the two groups: 1. The experimental or LIPUS group (LIPUS and clear aligners) 2. The control group (clear aligners). The Aevo System was offered to patients to improve their orthodontic treatment experience, and all patients in the experimental group chose to use the device voluntarily.

**Fig. (1):**
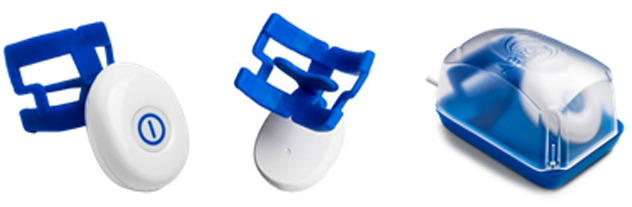
Aevo System (Model A3-0000) and accessories retrieved from SmileSonica Inc. website (https://www.smile sonica.com/products/aevo-system).

The Aevo System is a LIPUS medical device tailored for use with either clear aligners or fixed braces. It is a battery-operated, portable device that can be used at home for 20 minutes daily. The Aevo System A3 specifications include an average ultrasound intensity of 30 mW/cm^2^, a 1.5 MHz operating frequency, amplitude modulation with a 1 kHz repetition rate, and a pulse pattern of 200 µs ON and 800 µs OFF (Fig. **1**).

Parameters were set at a 0.05 significance level and 80% power for sample size determination. At first, the calculations suggested a required sample size of 59 patients per group to detect an anticipated 49% outcome difference based on prior studies. Practical constraints led to the selection of 34 patients per group, totaling 68 participants.

• LIPUS Group

• N = 34

• Clear aligners + Aevo System

• Control Group

• N = 34

• Clear Aligners

Patients included in the study were those aged 12 or older, treated exclusively with clear aligners or with clear aligners in conjunction with the Aevo System A3, with at least an adherence rate of 67%. Patients were also required to have good oral hygiene, no active periodontal disease, and comprehensive records, including radiographs, photographs, scans, treatment cards, session logs, and Invisalign data. Patients with significant medical or dental issues, those receiving extraction orthodontic treatments, having extra teeth at the start of treatment, TMJ disorders, tooth movement conditions, involvement in other clinical studies, or failure to comply with aligner wear time were excluded.

Based on prior studies, adherence thresholds were set at 85% for aligners and 67% for LIPUS, reflecting effective treatment outcomes at these levels. The 85% aligner threshold ensures adequate wear time to maintain progress [29], while the 67% LIPUS threshold aligns with studies showing significant treatment time reduction [6]. The lower age limit was considered at 12 years, regarding the typical eruption of permanent teeth and sufficient skeletal maturity necessary for effective clear aligner therapy [30]. Extraction cases were excluded to maintain a uniform sample, avoiding healing and tooth movement variables that could confound the study's focus on LIPUS with clear aligners [31].

Data collected retrospectively included demographic details (patient ID, group, sex, age), adherence with the Aevo System, and American Board of Orthodontics Discrepancy Index (ABO DI) scores pre-and post-treatment (Sager guidelines were followed). Criteria for successful treatment completion were proper alignment and occlusion, resolution of dental issues, patient satisfaction with the outcome, and a post-treatment ABO DI score under 15, indicating the attainment of treatment goals [32].

The bone radio density was measured before and after orthodontic treatment using CBCT scans captured by the iCAT^®^ scanner (Imaging Sciences International in Hatfield, Pennsylvania, USA). The scans were characterized by specific parameters: a width of 16 cm, height of 13 cm, 120 kVp, 24 m As, a scan time of 20 seconds, voxel size of 0.3 mm, and 303 basis projections.

InVivo Dental 5.0 software by Anatomage Inc., based in San Jose, CA, USA, was utilized to assess bone radiodensity. This software allowed for setting standardized sagittal and coronal views to measure Hounsfield units accurately across predetermined alveolar bone locations.

The calibration involved rotating the image to align the posterior rim of the incisive foramen with the posterior nasal spine within the same slice, as depicted in Fig. (**2**). For the upper arch, the coronal section alignment was established at the level of the posterior rim of the incisive foramen, as shown in Figs. (**3** and **5**). The lower arch was adjusted to the midpoint line passing through the posterior inferior point of the second cervical vertebra, illustrated in Figs. (**4**-**6**).

To control for confounding factors, data from the experimental group were matched with the control group based on age, sex, and treatment complexity according to ABO DI scores. Statistical analyses were conducted using JASP software, with group differences evaluated through paired *t*-tests and Mann-Whitney U Tests, setting significance at p < 0.05.

This research examines the impact of LIPUS on bone radio density during clear aligner orthodontic treatment. Enhancing bone remodeling and stability, LIPUS may lead to more predictable and efficient orthodontic outcomes, reducing treatment time and refinements. This could lower costs and improve patient comfort. Additionally, it holds potential benefits for patients with compromised bone health, offering personalized treatments that may improve long-term results and overall quality of life.

## 3 RESULTS

This study investigates the use of LIPUS to enhance bone radio density during treatment, hypothesizing that it could improve bone remodeling and may reduce relapse risk. This study investigated the records of 34 individuals in each group. The control group comprised 22 females and 12 males (64.71% *Vs*. 35.29%), with a median age of 24.6 years and an average age of 29.85 ± 14.85 years. The LIPUS group included 19 females and 15 males (55.88% vs 44.12%), with a median age of 33.5 years and an average age of 36.29 ± 12.78 years. Both groups were matched in their age, gender, and complexity. A comparison of ages between groups (independent samples t-test, p=0.05) resulted in no substantial age difference. Additionally, no significant correlation between group placement and sex distribution was observed (chi-square test, p=0.620).

The distribution of malocclusion classes was identical between the LIPUS and control groups: Class 1, 55.88%, Class 2, 23.53%, and Class 3, 20.59%. Each group had a balanced distribution of treatment complexities, with five mild cases: ABO DI range 1-10, 11 moderate cases: ABO DI range 11-20, 11 complex cases: ABO DI range 21-30, and 7 very complex cases: ABO DI range 31-100.

Pre-treatment ABO DI scores were similar, with the Control group averaging 22.88 ± 11.92 *vs*. 22.09 ± 11.55 for the LIPUS group. Post-treatment results were also alike, with the Control group at 4.76 ± 4.39 *vs*. 4.74 ± 3.29 for the LIPUS group. No significant difference in post-treatment scores between the groups was observed (independent samples t-test, p=0.67), suggesting equivalent effectiveness in achieving orthodontic outcomes.

In the control group, analysis of bone radio density measurements in the alveolar bones revealed minor changes post-treatment. In the upper arch (maxillary alveolar bone), the mean pre-treatment bone radio density stood at 657.4 HU, slightly decreasing to 650.5 HU post-treatment, with a paired t-test yielding a p-value of 0.86, indicating no significant change. Similarly, in the lower arch (mandibular alveolar bone), the mean pre-treatment density was 836.7 HU, which decreased to 828.1 HU post-treatment, and a p-value of 0.83 also suggested no significant change (Table **1**).

In the LIPUS group, there were notable changes. The upper arch (maxillary alveolar bone) showed a significant increase from a pre-treatment mean of 444.6 HU to 751.3 HU post-treatment (p <0.001). For the lower arch (mandibular alveolar bone), the pre-treatment density was 767.7 HU, which increased to 823.4 HU post-treatment; however, this increase was not statistically significant (p= 0.17) (Table **2**).

## 4 DISCUSSION

Stability after achieving the desired orthodontic treatment is a challenge. The effect of LIPUS on bone radio density post-orthodontic treatment was examined in this study, hypothesizing that LIPUS possibly enhances bone formation and hence supports stable outcomes. Our results show a significant increase in bone radio density in the LIPUS group compared to the control group, as evidenced by higher Hounsfield units in the maxillary alveolar bone, which is crucial for maintaining stable tooth positioning and reducing relapse risk.

As shown in Table **1**, bone radio density in the control group decreased slightly post-treatment, with no significant changes in the upper or lower arches. In contrast, Table **2** highlights the substantial increase in bone radio density in the LIPUS group in the upper arch, particularly in the maxillary alveolar bone. The changes observed in the lower arch were not statistically significant, indicating that LIPUS might have a more pronounced effect on the upper arch. Figs. (**7** and **8**) show the mean changes in Hounsfield units over time for both the control and LIPUS groups.

These findings agree with previous research indicating that LIPUS enhances bone remodeling and density [27, 33].

Fu Zheng *et al*. found that LIPUS facilitated osteogenic processes by modulating mechanosensitive ion channels in periodontal ligament cells [27]. Another study found that LIPUS enhances osteogenic processes, supporting the notion that LIPUS can improve orthodontic treatment [33]. In the study of Miura and colleagues, the authors observed notably reduced mobility in mini-screws treated with LIPUS, indicating that LIPUS may lower the likelihood of mini-screw loosening during orthodontic treatment. It can link to enhanced bone mini-screw contact and faster bone formation and remodeling, leading to more stable orthodontic results [20]. El-Bialy *et al*. (2003) reported that therapeutic ultrasound increases mandibular condylar growth in rabbits through enhanced endochondral bone formation [34]. The study of Kaur and El-Bialy showed an elevation in osteogenic markers, including RANK-L and OPG, which are crucial for bone remodeling and faster tooth movement [6]. These studies support the increased bone radio density observed in the LIPUS group.

Santosh *et al*. (2020) described LIPUS parameters enhancing alveolar bone remodeling by increasing the expression of osteogenic markers such as Interleukin-8 and BFGF [35]. Pascoal *et al*. (2024) found that particular ultrasound stimulation settings significantly boost metabolic activity and the expression of osteogenic markers in osteoblasts and periodontal ligament fibroblasts [28]. This underscores the importance of optimizing LIPUS protocols for achieving consistent clinical results.

Xue *et al*. (2013) found that LIPUS significantly increases orthodontic tooth movement (OTM) in a rat model through the BMP-2 signaling pathway and enhanced RANKL expression, promoting alveolar bone remodeling [36]. Yuri Higashi *et al*. (2020) showed that increasing LIPUS application frequency enhances osteoclast differentiation, potentially accelerating tooth movement by modulating osteoclast activity [37].

Tanaka *et al*. emphasized LIPUS's mechanisms promoting cellular responses for accelerated tissue repair and regeneration [38], consistent with the enhanced bone radio density observed in our LIPUS group. El-Bialy discussed LIPUS's mechanistic pathways, such as activating mechanosensitive ion channels and integrating integrins to enhance bone remodeling [39]. Zheng *et al*. (2024) reported that LIPUS downregulated Piezo1, enhancing osteogenesis under mechanical stress [27].

Qin *et al*. (2023) indicated that LIPUS mediates tissue regeneration and reduces inflammation through macrophage polarization [40]. However, Bahammam and El-Bialy (2022) showed that LIPUS did not significantly affect bone thickness or height in maxillary expansion with clear aligners, suggesting variability in clinical effectiveness depending on conditions and protocols [41]. Qamruddin *et al*. (2015) reviewed animal studies showing that LIPUS accelerates tooth movement by influencing cellular activities and bone remodeling [42].

El-Bialy *et al*. highlighted LIPUS's role in facilitating orthodontic tooth movement and tissue repair [39]. They reported enhanced cellular responses, including upregulated osteogenic markers such as RUNX2 and osterix [39], aligning with our enhanced bone radio density observations in the LIPUS group. Toy *et al*. found that LIPUS treatment increased VEGF and osteocalcin immunoreactivities, suggesting higher osteoblastic activity and accelerated orthodontic treatment [43]. Zhou *et al*. (2023) found that LIPUS enhances osteogenic differentiation and modulates bone homeostasis via the EphrinB2/EphB4 signaling pathway [26].

Orthodontic tooth movement involves bone remodeling regulated by the TNF receptor-ligand family, including OPG, RANK, and RANK-L. Mechanical stress induces RANK-L release, promoting osteoclast differentiation and activity. LIPUS potentially influences orthodontic tooth movement through mechanical strain on cell membrane receptors, activating cellular signaling pathways like FAK, MAPK, and Rho, enhancing bone formation, angiogenesis, and osteoblast differentiation [44-47]. Alazzawi *et al*. (2018) found that LIPUS enhances tooth movement velocity and bone remodeling during orthodontic tooth movement in rats, highlighting molecular pathways involved in accelerated orthodontic procedures [48]. Yingying Wang *et al*. (2024) showed LIPUS efficacy in improving peri-implant osteogenesis under diabetic conditions, suggesting its potential in challenging orthodontic cases [49].

Moreover, the preliminary findings of this study indicate that the LIPUS group exhibited lower Hounsfield Unit values in bone radio density before treatment in both the maxillary and mandibular arches compared to the Control group. This variation could be linked to age-related differences in bone radio density, as bone density tends to diminish with age [50].

## STUDY LIMITATIONS

We aimed to evaluate bone quality; however, using Dual-Energy X-ray Absorptiometry (DEXA) to measure bone density would have been ideal [51]. Instead, due to the limitation of a retrospective study, we used cone-beam computed tomography (CBCT) and measured bone radiodensity. This approach has limitations, as gray values from CBCT cannot be directly converted to Hounsfield units due to the lack of clinical studies and standardization of CBCT scanners [52].CBCT scans are not calibrated in the same way as traditional computed tomography (CT) scans, and the conversion of gray values to Hounsfield units can introduce errors due to variations in scanner settings, patient positioning, and the specific software used for analysis. Several studies have highlighted that the accuracy of this conversion depends on the specific calibration of the CBCT system and the tissue type being measured, with bone density measurements being less accurate than those obtained from CT scans [52]. These limitations should be considered when interpreting the results of this study, as they may affect the precision of the bone radio density measurements.

The study's retrospective design further limits establishing a direct cause-and-effect relationship. This design was selected due to the availability of pre-existing patient records from a private orthodontic clinic. Retrospective studies are often more feasible in clinical settings where data have been collected, allowing for the analysis of LIPUS’s effects without the logistical challenges of conducting a prospective study. While a prospective design would provide stronger evidence regarding causality, the retrospective design of this study offers valuable insights into the effects of LIPUS on bone radio density during orthodontic treatment with clear aligners.

Additionally, the small sample size and the single-clinic evaluation limit the generalizability of our findings. This happened due to the recent release of the Aevo System 3.0 device in 2019, which posed further challenges, as it takes time for orthodontic treatment to be completed, and finding compliant patients with all the necessary records available was difficult. The small sample size may impact the findings' statistical power and the results' generalizability to broader populations. The relatively small sample size may have reduced the ability to detect subtle differences between the groups, particularly in the lower arch, where the changes in bone radio density were not statistically significant. Future studies could consider expanding the sample size to enhance statistical power and increase the representativeness of the findings. The recruitment criteria for this study, which included patients aged 12 or older with a minimum adherence rate of 67% to the treatment protocol, aimed to ensure a homogeneous sample, but further research could include a more diverse participant pool to improve the generalizability of the results.

Furthermore, CBCT was already acquired, and due to the study's retrospective nature, we had access to CBCT images, not patients, to get DEXA. The lack of detailed reliability analyses and the exclusion of extraction cases also limit the broader applicability of the findings.

Future research should focus on larger sample sizes and multi-center studies to enhance the generalizability of the findings. Additionally, prospective studies using DEXA for bone density measurements alongside CBCT could provide more accurate assessments and comparisons. Prospective, double-blinded randomized controlled trials (RCTs) and exploring the impact of LIPUS on orthodontic relapse and retainer fit post-treatment would also provide a more comprehensive understanding of its long-term benefits.

This study highlights the positive potential of LIPUS on bone radio density following orthodontic treatment with clear aligners. LIPUS showed that it could be promising in enhancing bone radio density, which is crucial for maintaining stable tooth positioning and reducing the risk of relapse. The preliminary findings can potentially support the hypothesis that LIPUS promotes bone remodeling, making it a valuable adjunctive therapy in orthodontics. These preliminary results are particularly relevant for patients with compromised bone health, such as osteoporosis.

Future research should aim to elucidate the molecular mechanisms by which LIPUS influences bone density and confirm these findings through larger, more diverse studies. DEXA should be used to measure bone density for more accurate assessments. The potential of LIPUS to enhance treatment outcomes and patient care in orthodontics warrants further investigation through prospective, double-blinded, randomized controlled trials (RCTs) and multi-center studies.

In addition, recent advancements in medical imaging and diagnostic technologies, such as interactive segmentation [53] and AI-based diagnostic recommendations [54], could further enhance the accuracy of bone density assessments in orthodontics. Moreover, incorporating deep learning models used in medical image segmentation [55] may improve the analysis of CBCT scans, offering new avenues for optimizing orthodontic treatment and stability.

Based on this study, it might be recommended that LIPUS be incorporated into clinical protocols to enhance bone remodeling, improve treatment stability, and potentially reduce relapse. Standardizing LIPUS treatment parameters across orthodontic practices will also ensure consistent and optimal outcomes. Policymakers might also consider advocating for including LIPUS in orthodontic insurance coverage to make it accessible to a wider range of patients, particularly those with compromised bone health.

## CONCLUSION

This study suggests that LIPUS enhances bone radio density, particularly in the upper arch, during orthodontic treatment with clear aligners. The findings support LIPUS as a potential adjunct for improving bone remodelling and stability in orthodontic treatments, which could lead to more predictable and stable patient outcomes. Clinically, orthodontists may consider incorporating LIPUS into treatment protocols, particularly for patients with lower bone radio density or those undergoing accelerated orthodontic treatments. While the lower arch showed minimal changes, these findings suggest that LIPUS may have a more pronounced effect on the upper arch. Further research with larger samples and more precise measurement methods, such as DEXA, is needed to confirm these results and investigate the long-term benefits of LIPUS in orthodontics. Additionally, Future research should focus on investigating specific hypotheses that arose during this study, including the potential for LIPUS to enhance retention outcomes and prevent relapse in orthodontic patients. Furthermore, studies could examine the effects of LIPUS on different patient groups, such as those of varying ages or those with compromised bone density conditions like osteoporosis.

## Figures and Tables

**Fig. (2):**
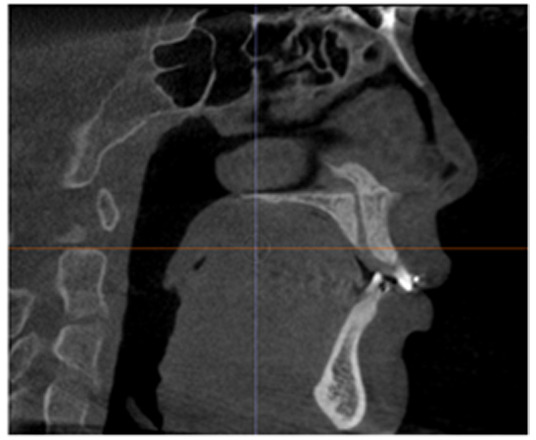
Sagittal and Coronal section showing the posterior rim of the incisive foramen and the posterior nasal spine (PNS). The posterior rim of the incisive foramen is the rear boundary of the bony canal in the maxilla. The posterior nasal spine (PNS) is a bony projection at the back of the nasal cavity.

**Fig. (3):**
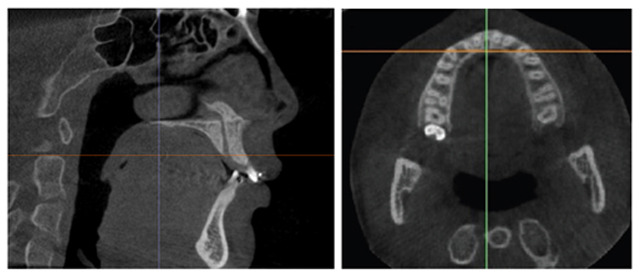
Reference lines for measuring the maxillary alveolar bone radio density passing through the posterior rim of the incisive foramen in axial and sagittal view.

**Fig. (4):**
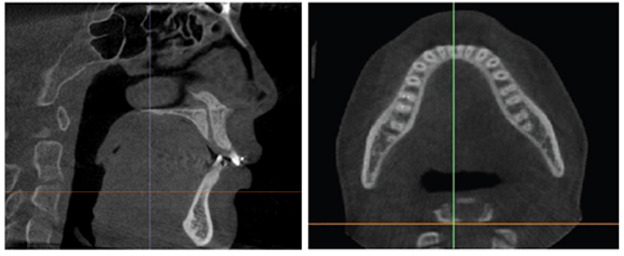
Reference line for measuring the mandibular alveolar bone radio density passing through the posterior inferior point of the second cervical vertebra in axial and sagittal view. Measurements of bone radio density were taken in the standardized coronal slices of both arches, specifically at the midpoint between the buccal and lingual cortical plates within the cancellous bone at five defined locations: bilaterally between the canine and lateral incisor, bilaterally between the lateral and central incisor, and directly at the midline, as indicated before and after treatment in Fig. (**5**) for maxilla and Fig. (**6**) for mandible.

**Fig. (5):**
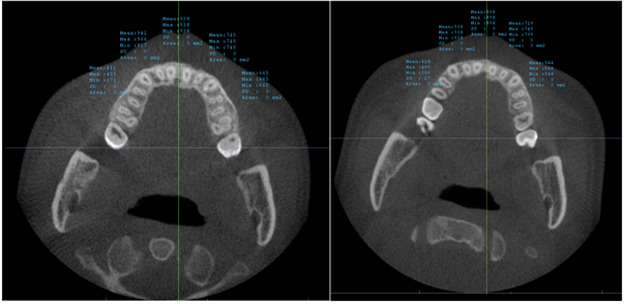
Measures the bone radio density at five locations in the Maxilla alveolar bone: between the right canine and lateral, right lateral and central, left central and lateral, left lateral and canine, and at the midline. The right image shows the bone radio density measurements before treatment, and the left image shows the bone radio density measurements after treatment.

**Fig. (6):**
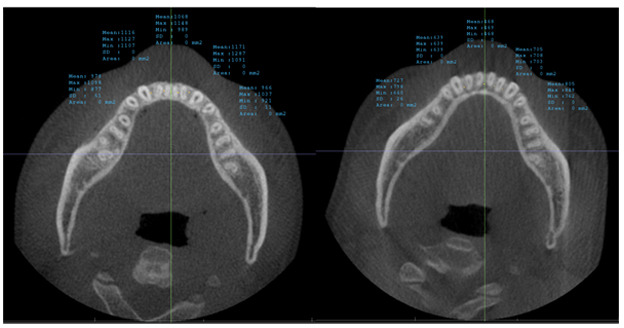
Measures the bone radio density at five locations in the Mandible alveolar bone: between the right canine and lateral, right lateral and central, left central and lateral, left lateral and canine, and at the midline. The right image shows the bone radio density measurements before treatment, and the left image shows the bone radio density measurements after treatments.

**Fig. (7):**
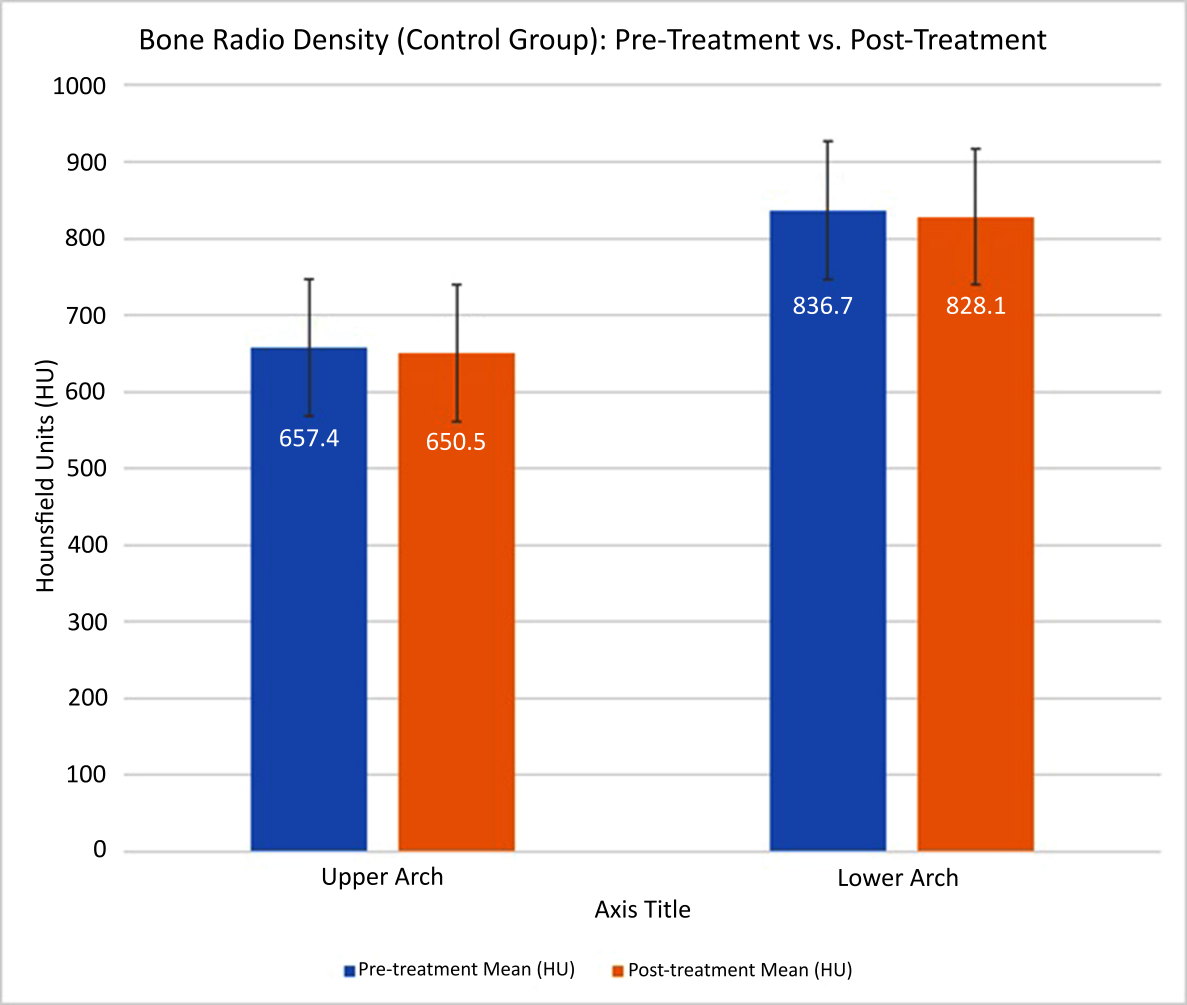
Bone radio density (hounsfield units) for the control group (pre-treatment *vs*. post-treatment) in the upper and lower arches. The graph compares the mean bone radio density (HU) before and after orthodontic treatment with clear aligners in the Control group.

**Fig. (8):**
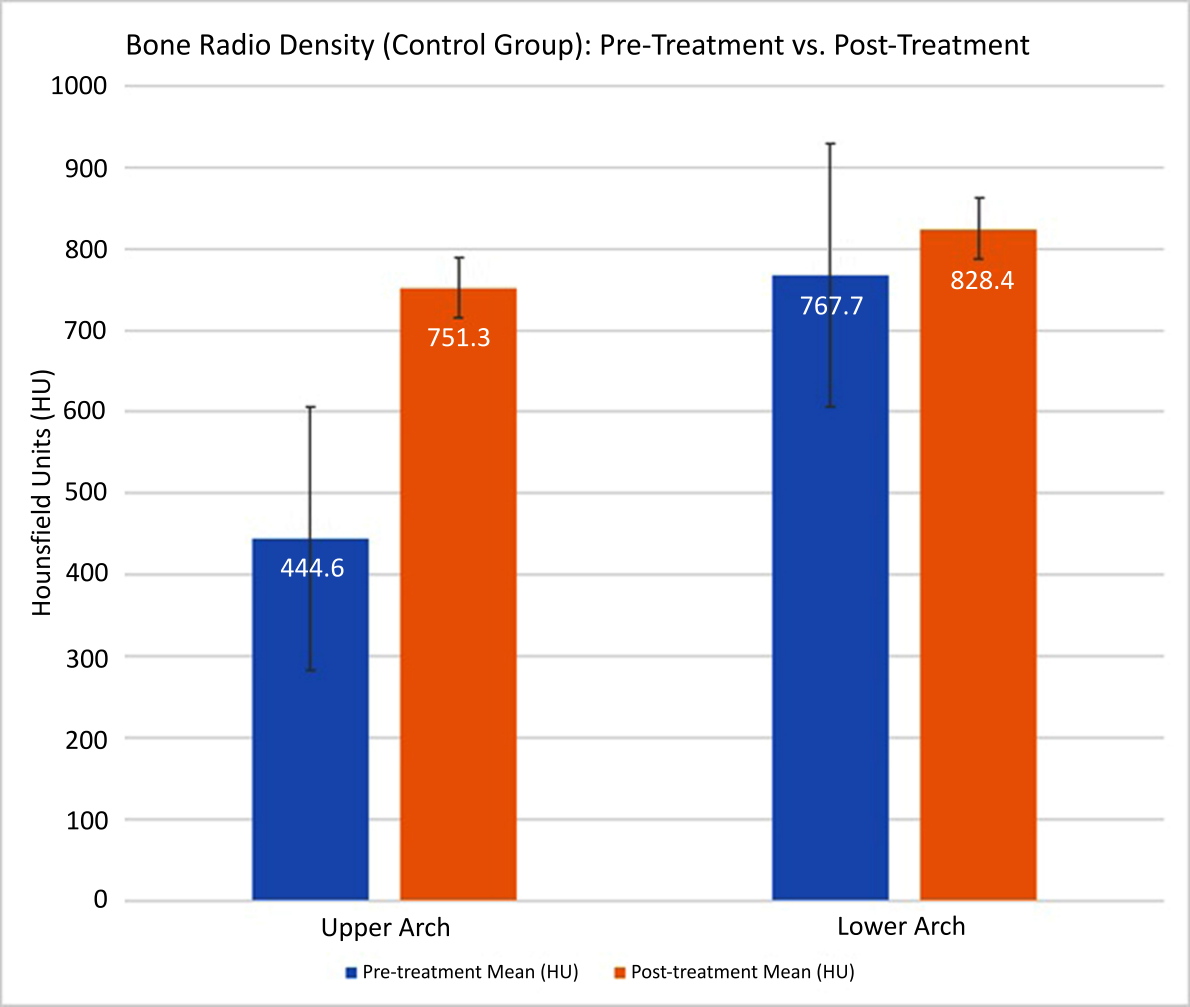
Bone radio density (hounsfield units) for the lipus group (pre-treatment *vs*. Post-treatment) in the upper and lower arches. The graph compares the mean bone radio density (hu) before and after orthodontic treatment with clear aligners in the LIPUS group.

**Table 1:** Bone radio density measurements in the control group.

Control				
Pre-treatment	T1 Mean (HU)	T1 Max (HU)	T1 Min (HU)	T1 SD (HU)
Upper Arch	657.4	702.0	616.1	8.1
Lower Arch	836.7	917.8	775.6	23.4
Post-treatment	T2 Mean (HU)	T2 Max (HU)	T2 Min (HU)	T2 SD (HU)
Upper Arch	650.5	695.4	614.7	8.0
Lower Arch	828.1	893.9	766.8	22.7
Upper Arch	P=0.86			
Lower Arch	P=0.83			

**Table 2:** Bone radio density measurements in lipus group.

LIPUS				
Pre-treatment	T1 Mean (HU)	T1 Max (HU)	T1 Min (HU)	T1 SD (HU)
Upper Arch	444.6	513.6	386.4	18.1
Lower Arch	767.7	842.6	701.8	22.9
Post-treatment	T2 Mean (HU)	T2 Max (HU)	T2 Min (HU)	T2 SD (HU)
Upper Arch	751.3	821.6	684.3	21.6
Lower Arch	823.4	882.9	763.9	16.4
Upper Arch	P < 0.001			
Lower Arch	P=0.17			

## Data Availability

The data and supportive information are available within the article.

## LIST OF ABBREVIATIONS

LIPUS: Low-Intensity Pulsed Ultrasound
OITRR: Orthodontically Induced Tooth Root Resorption
CBCT: Cone Beam Computed Tomography
OPG: Osteoprotegerin
ALP: Alkaline phosphatase
ABO DI: American Board of Orthodontics Discrepancy Index

## STANDARDS OF REPORTING

STROBE guidelines were followed.
